# Assessing Knowledge and Practices of the Community towards Corona Virus Disease 2019 in Mbale Municipality, Uganda: Across Section Study

**DOI:** 10.24248/eahrj.v5i1.647

**Published:** 2021-06-11

**Authors:** Naziru Rashid, Aisha Nazziwa, Rehema Kantono, Hassan Kasujja, Swaibu Zziwa

**Affiliations:** a Islamic University in Uganda

## Abstract

**Background:**

The Corona virus disease, first identified in Wuhan city, Hubei province of China, is a respiratory illness caused by Novel Corona Virus also known as Severe Acute Respiratory Syndrome Corona Virus 2 (SARS Cov.2). The disease is characterised by; dry cough and shortness of breath with difficulty in breathing and at least 2 of the following; fever, chills, muscle pain, headache, sore throat and loss of test and smell. Uganda in general and Mbale in particular has people of diverse culture, religion and ethnic background as well as diverse socio economic activities with various practices. This multi-cultural environment creates differences in perception of information and practices. Most cultures encourage socialisation through social functions like attending weddings, funerals, work places and gatherings and Muslims who have to go for congregation prayers in the mosques 5 times a day among others. This puts such communities at risk of spreading the disease very fast and slow in adapting to control measures

**Aim:**

In this study, we aimed at assessing knowledge and practices of the community towards COVID 19 in Mbale municipality.

**Methods and Materials:**

A cross section study was used; Data was obtained using a Questionnaires to a sample of 355 respondents and an observation tool was also used to observe behaviour patterns and practices of 776 participants towards the control measures of COVID-19.

**Results:**

There was a total of 355 respondents with 208/355 (58.59%) male and 147/355 (41.4%) female. 149/355(42%) possessed good knowledge, 131/355(36.9%) had moderate knowledge and 75/355(21%) had a little knowledge on COVID-19. Participants who were single and aged between 21–30 years were found to be more knowledgeable than other groups (*P value=.001* and *P value=.003* respectively). The source of COVID 19 information was mainly from television and radios 124/248 (50%) and social media 34/248 (21.8%) and the least source of information being 14/248(5.6%) and 9/248(3.6%) from health workers and Religious leaders respectively. 496/776 (64%) of the respondents observed, washed their hands and only124/776 (16%) of the respondents wore face masks. 98/776 (12.6%) were seen shaking hands and 15/776(2%) were seen hugging.

**Conclusion:**

Use of appropriate and well-designed Health education materials on radios, televisions and social media platforms like Facebook and twitter among others can be effective means of communication since they can reach the highest number of people. Ministry of Health should design ways for systematically integrating both political and religious leaders in Health Education Campaigns. Government should provide facemasks and enforce their use. A study to assess the ability of both political and religious leaders in health promotion campaigns should be carried out.

## BACKGROUND

The Corona virus (COVID-19) disease first identified in Wuhan city, Hubei province of China, is a respiratory illness caused by the Novel Corona Virus also known as Severe Acute Respiratory Syndrome Corona Virus 2 (SARS-CoV 2).^1^ The disease is characterised by dry cough and shortness of breath with difficulty in breathing and at least 2 of the following; fever, chills, muscle pain, headache, sore throat and loss of taste and smell.^4,5^

It was declared a public health emergency of international concern and a global pandemic by the World Health Organization (WHO) on 30^th^ January 2020 and 11^th^ March 2020 respectively.^2,3,4^

There is still no known specific cure for COVID-19 apart from the vaccines for Immunisation. The World Health Organization (WHO) and the local authorities have guided on a number of infection control measures among Communities including; hand washing, hand sanitising, physical distancing, observing respiratory hygiene, avoiding hand shaking and hugging among others.^11^ Many communities tend to live a usual life which puts their lives at risk of contracting the disease. There are different sources of information about COVID-19 within communities. In Uganda, the sources of information includes Main stream media, politicians, religious leaders, cultural leaders, celebrated personalities and Social media platforms among others. There has also been installation of measures based on their known public health impact, majorly social distancing, wearing facemasks and hand washing in public places as well as a total country lockdown to increase on the social distancing, These same measures have been instituted in other African countries.^8^

These Non-Pharmaceutical Interventions (NPI) are very impactful strategies in delaying disease transmission and they reduce the impact of the disease on the country's health care systems, especially in resource limited settings like Uganda.^7^ In such circumstances, the success in controlling the spread of disease largely depends on how the community responds to and observes the public health measures put in place by the government's respective authorities. This is also largely dependent on the knowledge and attitude towards the disease, the source of health information and the rationale behind the measures put in place.

Uganda has people of diverse culture, religion and ethnic backgrounds with various practices. This multi-cultural environment creates differences in perception of information and practices. Most cultures encourage socialisation through social functions such as weddings funerals, work places, attending sports gatherings and Muslims have to go for congregation prayers in the mosques 5 times a day.

This Diversity is seen in Mbale District due to its unique and strategic location. Mbale district is located along the high way that joins Kenya to South Sudan and the Democratic republic of Congo through Uganda, this therefore, attracts many business people as well as administrative offices in the region. In addition, the traditional cultural dance known as “Kadodi” that brings people together during the tradition cultural public circumcision ceremony known as “Imbalu” puts the people of Mbale and other neighbouring districts at a higher risk than other districts. Such practices put the community at risk of not only spreading the disease, but also being slow on adopting to infection control measures.

Behaviours and perceptions in communities are drivers of spread, control and management of Infections.^12^ It is noted that during a public health crisis, like COVID-19, misinformation spreads faster than the Disease itself.^9^ For instance, most people in western Uganda largely believed in myths and rumours like; COVID-19 only affects the whites and not Africans. They also believed that taking of alcohol can prevent the spread of COVID-19.^9,10^ Such beliefs, myths and misinformation can affect the efforts by the Government authorities to control the spread of COVID-19. The effectiveness of such measures depends on an individual's degree of involvement and adherence to control measures.^13,14^ The willingness to adopt to new changes in their day-to-day behavioural activities (like hand shaking, hugging, etc.) largely depends on people's perceived risk of contracting the disease in question severity or impact of the disease on their lives. This also depends on people's level of awareness and knowledge about the disease. To date, there is no documented study about the level of knowledge, practices and responses of Ugandan Communities towards COVID-19.

Appreciating the role of human behaviour in mitigating the spread of communicable diseases, in this study we therefore assessed the level of knowledge of COVID-19, the Practices and responses of the Community towards COVID-19 and the source of information. Our Findings will enable government through the Ministry of Health (MoH) to design appropriate message for Health Education through mass media and social media, design programs for training of different leaders to Health educate communities and appropriate implementation of the control measures especially in the Post Lockdown period.

## METHODOLOGY

### Study Setting and Study Design

This was a cross-sectional study conducted between 1^st^ April and 30^th^ May 2020 within villages and towns of Mbale municipality, Mbale district. Mbale district is located in the Eastern region of Uganda in East Africa, approximately 225km (140miles) North East of the capital Kampala.^15,16^

By 2019, the district was estimated to have 568,000 people, 52.3% being females.^15,16^ The main economic activities being farming and trade (business).

The district has 12 Government dispensaries (Health Centre level II), 17 health centres (level III) at county, 4 health centres (level IV) at sub-district with 2 hospitals. More so, it has 4 private/NGO dispensaries (level II), 7 health centres (Level III) and no hospital. There is Regional Referral Hospital with 332 beds.^19^

Unlike other towns in Uganda, Mbale Municipality specifically and Mbale District in general have a unique location being on the high way joining Kenya to south Sudan and the democratic republic of Congo through Uganda. The district hosts a number of administrative offices and businesses and thus brings a large population of people together. This puts the people within the district at a higher risk of contracting the disease.

### Study Population Inclusion criteria

All adult male and female who consented to participate in the study were included in the study.

### Exclusion Criteria

During the process of the study, all those who declined at the beginning or during the process of data collection to participate were excluded from the study. Children below the age of 16 years were excluded from the study

### Sample Size

A total of 350 participants were enrolled into the study. This sample size was obtained using the formula for calculating sample size.

$$\text{N}=\frac{\text{Z}2\text{PQ}}{\text{δ}2}$$

Where N is the sample size that we are looking for.

Z is the standard normal deviation at 95% Confidence equal to 1.96

P is estimated Prevalence or Proportion of people informed about the disease, for our case we used a conservative value of 50%, because no study has estimated a prevalence in Uganda and other countries with the same socio demographic area like Uganda.

Q is 100% - P will be equal to 100-50 equal to 50%

E is the maximum acceptable error and was taken as 0.05 Required sample size is 392.

This was obtained mathematically as follows

(a) 2ZPQ=(2×1.96×50/100×50/100)=0.98

(b) δ2=(0.05×0.05)=0.0025

(a) / (b) = 0.098/0.002 = 392 Participants

### Sampling Procedure

Participants were randomly sampled using the convenient sampling method. A pre-tested Questionnaire was used. Pre-testing was done on 16 participants at Islamic University in Uganda (IUIU), Health Centre, IUIU department of Mass communication, IUIU main gate entrance and at IUIU Mosque.

Research Assistants were IUIU health Centre staff and Mass communication final year students. Participants were mad to sign consent forms, taken through the questions using either English, Luganda or lumsaba (Lugisu languages) which are the local languages in the area to enable participants give their appropriate responses. The whole process could take 15 to 25 minutes on average.

For the observations a data collector made observations and recorded behavioural patterns of the participants in the community.

Observations took place at key areas where large concentrations of people are expected, these included; Nkoma Market Main entrances, Kikindu Market Main entrances, Abrah Shopping Centre, Bam Shopping Centre, Mbale taxi park station, Mbale Bus Park station and Islamic university In Uganda (IUIU) main entrance, all located in Mbale Municipality.

Observers could spend between 2 to 3 hours at each point for 3 alternating days in a week.

### Variables

Dependent variables included; knowledge, attitude and practices of the community towards Novel Corona Virus. The independent variables included; Socio-demographic variables, likeAge, Sex, marital status, education, occupation, and income.

### Data Analysis

Descriptive statistics of the variables were computed as Mean ±SD and frequencies (n, %). The relationships between the categorical variables were investigated by using Pearson's Chi-Square test. Also, independent-samples, t-test were used to investigate the difference between groups with regard to numerical variables. In all calculations, *P<.05* was considered as the level of statistical significance. Statistical analysis was performed using SPSS 20 (IBM SPSS statistics, Somers, NY)

### Anonymity

Participants’ identification information was not included anywhere in the data collection tool

### Informed consent

All participants were made to sign an informed consent document before participating in the study and were allowed to withdraw from the study at stage of data collection. Permission to conduct the research was given by the Research Coordination Committee (RCC) of the Islamic University in Uganda with reference number **RCC/FHS/20/001**

### Study Limitations

355 of the required 392 participants were recruited. This was because of the restrictions of movements and transport as a result of the country wide lockdown during the process of data collection.

## RESULTS

### Socio-Demographic Characteristics of the Study Respondents

There was a total of 355 respondents, with 208 (58.59%) males and 147 (41.4%) females. More than half, 292 (82.25%) of the respondents were below 40 years old and 121/350 (34.57%) had Secondary Education, 109/350 (31.14%) had primary Education 75/350 (21.42%) had Tertiary education and the rest 45/350 (12.86%) were not educated at all. Majority of the respondents 134/354 (37.85%) were Muslims followed by 93/354 (26.27%) Catholics and 67/354 (18.92%) Anglicans. 115/347 (33.14%) of the respondents had businesses, 42/347 (12.10%) were involved in farming, and 81/347 (23.34%) had employment. (Table 1).

**TABLE 1:** Social Demographic Characteristics and Comparison with Knowledge Score

	n(%)	Knowledge score mean±sd	*P value*
**Age (years) n=249**			
21–30	111(44.5)	3.81±0.84	.001
31–40 years	75(30.1)	3.47±0.65	
41–50 years	33(13.2)	3.37±0.89	
above 50	30(12)	3.35 ±0.89	
**Marital status n=349**			
single	167(47.9)	3.84±0.86	.003
married	159(45.6)	3.56±0.78	
others	23(6.6)		
**Education level =350**			
not educated	45(12.9)	3.51±0.88	.082
primary level	109(31.1)	3.60 ±0.87	
secondary level	121(34.6)	3.75 ±0.81	
tertiary institution	75(21.4)	3.85±0.79	
**Religion n=354**			
Muslim	134(37.9)	3.64 ±0.88	.368
catholic	93(26.3)	3.77±0.76	
Anglican	67(18.9)	3.67 ±0.85	
Born again	47(13.3)	3.80 ±0.76	
others	13(3.7)	3.35 ±1.05	
**Occupation n=266**			
farming	42(12.1)	3.56±0.85	.181
business	115(33.1)	3.65 ±0.75	
employed	81(23.3)	3.6±4 0.78	
others	109(31.4)	3.8±3 0.95	

### Knowledge of the Mbale Residents towards COVID-19

Table 2 shows knowledge of Mbale residents towards COVID-19. Majority of the respondents, 70.9% agreed that they can differentiate between the symptoms of COVID-19 from flu. Knowledge score was at 3.37 ±1.355, and 250 (72.0%) agreed that COVID-19 can spread from one person to another with mean of 3.81.

**TABLE 2:** Knowledge of the Mbale Residents towards COVID-19

Question	agree	Not agree	Don't know	Mean Knowledge score
Q1. I know the causes of COVID-19 n=354	188(53.1%)	83(23.4)	83(23.4%)	3.35±1.307
Q2. COVID-19 can spread from one person to another n=347	250(72.0%)	51(14.7%)	46(13.3%)	3.90±1.135
Q3. I can differentiate the symptoms of COVID-19 from flue n=350	199(70.9%)	93(26.6%)	58(16.6%)	3.37±1.355
Q4. COVID-19 can spread from one person to another through handshake n=346	245(70.8%)	54(15.6%)	47(13.6%)	3.85±1.186
Q5. COVID-19 can be spread from one person to another by staying in close contact or gatherings n=349	257(73.7%)	46(13.2%	46(13.2%)	3.95±1.126

Fifty three percent (53%) of the respondents agreed that they know the causes of COVID-19, 23.4% did not know and the rest were not sure. Regarding COVID-19 disease transmission, 250/355 (72.1%) of the respondents agreed that COVID-19 could be spread from one person to another, 14.7% disagreed and 13.3% were not sure that COVID-19 could be spread from one person to another. COVID-19 can spread from one person to another through handshake. 70.8% agreed, 13.6 were not sure and 15.6% of the respondents disagreed that COVID-19 can spread from one person to another through handshake. 70.8% agreed, 13.2 were not sure and 15.6% disagreed that COVID-19 can be spread from one person to another by staying in close contact or gatherings.

Majority of the respondents agreed on whether COVID-19 can spread from one person to another through hand shake with a mean of 3.85 ±1.186, COVID-19 can spread from one person to another with mean 3.9 ±1.135 and COVID-19 can be spread from one person to another by staying in close contact or gatherings with mean 3.95 ±1.126.

### Comparison of Knowledge Score according to the Participants’ Age, Marital Status, Education, Religion and Occupation

We grouped level of knowledge into; high level of knowledge if a respondent scored mean ≥4, moderate level if score is mean ≥ 3.1 and mean ≤ 3.9 and low level if a respondent scored mean ≤ 3.

Table 2 shows that most of the respondents 149/355 (42.0%) possessed good knowledge, a considerable number 131/355 (36.9%) had moderate knowledge, while some of them 75/355(21.0%) had little knowledge on COVID-19.

Bivariate analyses of the level of knowledge was performed in relation to several independent variables: gender, age, education, occupation and marital status. Knowledge score was found to be statistically significant (P value=0.001) with Age group between 21-30 years being more knowledgeable than other age groups. Also, the knowledge score of those who are single was found to be significantly different from those who were married with (P value=0.003).

### Source of Information about COVID-19

**FIGURE 1:**
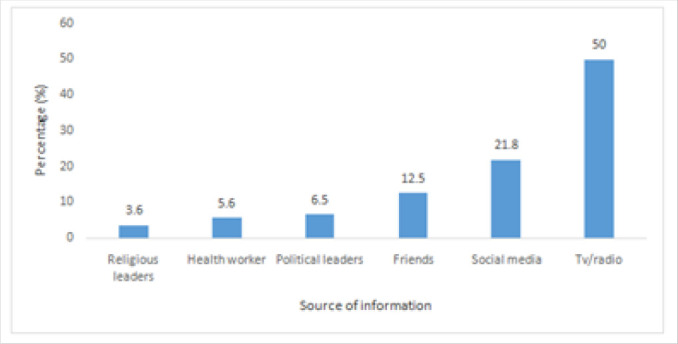
Source of Information about COVID-19

Figure 1, shows that among the participants who had knowledge about COVID-19, 24/248 (50%) got the information through Televisions and radios, 54/248 (21.8%) through social media, 16/248 (6.5%) from political leaders, 9/248 (3.6%) from religious leaders, 14/248 (5.6%) from health workers and 31/248 (12.5%) from friends.

### Practices of Mbale Residents towards COVID-19

To ascertain whether the guidelines for controlling COVID-19 were being implemented, 776 people were observed. The observations took place at key areas where large concentrations of people are expected, these included; Nkoma Market Main entrances, Kikindu Market Main entrances, Abrah Shopping Centre, Bam Shopping Centre, Mbale taxi park station, Mbale Bus Park station and Islamic university in Uganda (IUIU) main entrance, all located in Mbale Municipality.

While 496/776 (64%) of the people observed washed their hands or used a sanitiser, only 124/776 (16%) wore a mask. On the other hand, wearing a mask is someone's free will and not many officers are there to remind people. 15/776 (2%) of the people were seen hugging and 98/776 (12.6%) were observed to be shaking hands. Figures are summarised in table 3.

**TABLE 3:** Knowledge of Mbale Residents towards COVID-19

Statements	N	Mean	Std. Dev
S1. I know the causes of COVID-19	354	3.35	1.307
S2. I can differentiate the symptoms of COVID-19 from flue	350	3.37	1.355
S3. COVID-19 can spread from one person to another	342	3.81	1.191
S4. COVID-19 can spread from one person to another through hand shake	346	3.85	1.186
S5. COVID-19 can spread from one person to another	347	3.90	1.135
S6. COVID-19 can-be spread from one-person to another-by staying in close-contact or gatherings	349	3.95	1.126

## DISCUSSION

The purpose of this study was to determine the level of knowledge, attitude and practices of the community towards COVID-19 in Mbale Municipality.

Since the initial outbreak of COVID-19 disease in China, the disease has spread widely to various countries. According to the Uganda Ministry of Health (MoH) update on the 20th of April 2020, the number of COVID-19 cases rose to 10,484 in Uganda with the majority of cases being in Elegu district in Northern Uganda.

Many studies have reported the importance of knowledge and practice of community towards reducing the spreading rate of diseases during epidemics and pandemics.^17^ Lack of Knowledge contributes to undesirable attitudes and practices which leads to negative impacts on infection-control.

In this current study, majority of the respondents 173/355 (48.7%) and 114/355(32.1%) had moderate and good knowledge about COVID-19 respectively.

During the Middle East Respiratory Syndrome (MERS) Coronavirus outbreak, a similar level of knowledge was detected among health care providers in Uganda^15^ and United Arab Emirates (UAE).^16^ This can partly explain why Uganda was among the best country to contain the COVID-19 pandemic at the time. Unlike a study in Saudi Arabia^18^ where participants’ education, occupation, gender, were significantly associated with the level of knowledge of COVID-19. Also, a study conducted in Jordan found women to have less knowledge about COVID-19 than male.^18^ In the current study, singles and those aged between 21–30 years were found to be more knowledgeable.

Whereas a good number of observed participants 496/776 (64%) practiced hand washing and sanitising, only 124/776 (16%) were observed putting on Face masks, despite the fact that both measures are equally important in controlling the spread of COVID-19 and therefore, there is need for this to be re-emphasised.

The motivation for hand washing could have been the need for business owners to protect their businesses from being closed by government authorities. Businesses such as public transport, shopping malls, markets and academic institutions had installed hand-washing facilities at their premises.

On the other hand Facemasks were scarce and a bit expensive at the time, thus the majority of the people in the community could not afford to procure them. Thus, the need by the Governments’ Ministry of Health to provide free Face Masks to the citizens. The current study showed that the main source of information about COVID 19 was radio, television and social media and the least being religious leaders and health workers. Therefore, the Ministry of Health should design good health education messages for both Social Media and mainstream media so as to reach to the highest number of people in the community. The Ministry of Health should design programs of utilising both political and religious leaders in health promotion campaigns.

## CONCLUSION AND RECOMMENDATIONS

This study provides baseline data to the government for preventive measures and areas of emphasis especially in the control measures if the good results of containing the pandemic are to be maintained and consolidated.

Use of appropriate and well-designed Health education materials on radios, televisions and social media platforms like Facebook and twitter among others can be effective means of communication since they can reach the highest number of people. Ministry of Health should design ways of systematically integrating both political and religious leaders in Health Education Campaigns. Government should provide facemasks and enforce their use. Since the government responded to the COVID-19 outbreak by providing immediate preventive activities to Ugandans, there is need for a follow up study to assess whether the preventive measures and guidelines are still being followed. Another study to assess the ability of both political and religious leaders in health promotion campaigns should be carried out.
